# Evaluation of Molecular Assays for Rapid Diagnosis of Invasive Fungal Infections: Clinical Experience with the Commercial CandID and AspID Real-Time PCR Assays in Paraguay

**DOI:** 10.3390/jof12080567

**Published:** 2026-08-01

**Authors:** José Pereira, Melisa Florentín, Amiliana Pineda, Desiree Almirón, Gemma Johnson, Monserrat Aldama, Idalina Franco, Hernán Barrios, Olga Aldama, Fernando Arévalos, Meredith Brown, Tom M. Chiller, Diego H. Cáceres

**Affiliations:** 1 Center for Dermatological Diseases, National Leprosy Control Program, Ministry of Public Health and Social Welfare, San Lorenzo 2160, Paraguay; amilianapineda@hotmail.com (A.P.); monse_aldama@hotmail.com (M.A.); idafranco2011@gmail.com (I.F.); barrioshernan279@gmail.com (H.B.); olgaaldamao@gmail.com (O.A.); arevalosocamposfernando@gmail.com (F.A.); 2 Instituto de Previsión Social, Asunción 1209, Paraguay; desireealmiron@gmail.com; 3 IMMY, Norman, OK 73069, USA; gemma-johnson@immy.com (G.J.); meredith-brown@immy.com (M.B.); tom-chiller@immy.com (T.M.C.); 4 Emerging Diseases Research Group, Studies in Translational Microbiology and Faculty of Medical Sciences, Universidad del Rosario, Bogota 111711, Colombia; 5 Center of Expertise in Mycology Radboudumc/CWZ, 6500 HB Nijmegen, The Netherlands

**Keywords:** candidemia, invasive aspergillosis, molecular diagnostics, real-time PCR, Paraguay

## Abstract

Invasive fungal infections, including candidemia and invasive aspergillosis, remain significant causes of morbidity and mortality in at-risk patient populations. Rapid, accurate diagnosis is critical for timely antifungal therapy and improved clinical outcomes. We evaluated two commercial molecular assays, the CandID Multiplex PCR and AspID Real-Time PCR, for detection of clinically relevant *Candida* and *Aspergillus* species, respectively, in at-risk patients in Paraguay. The CandID assay demonstrated strong agreement with blood culture in detecting candidemia. The AspID assay showed high concordance with galactomannan testing, particularly in bronchoalveolar lavage specimens, supporting its role as a complementary diagnostic tool. Our findings underscore the value of integrating molecular diagnostics into routine fungal diagnostic workflows to improve early detection of invasive fungal infections.

## 1. Introduction

Invasive fungal infections, such as candidemia and invasive aspergillosis (IA), represent major diagnostic and therapeutic challenges, particularly in immunocompromised and critically ill patients [1,2,3,4,5,6]. *Candida* bloodstream infections (BSIs) are life-threatening and associated with high mortality, especially when diagnosis and treatment are delayed [3,4,7]. Conventional laboratory methods, including direct microscopic examination and culture, often require several days for identification and may lack sensitivity, particularly in patients with low fungal burden or prior antifungal exposure [1].

IA primarily affects immunocompromised individuals, including patients receiving solid organ transplantation (SOT), hematopoietic stem cell transplantation, or chemotherapy [6,8,9,10]. Early diagnosis is challenging due to nonspecific clinical symptoms and limitations of conventional diagnostic tools such as microscopy, culture, and antigen detection [11].

The development of molecular assays offers significant promise for the rapid and more sensitive detection of fungal pathogens directly from clinical specimens. Beyond conventional real-time PCR, several complementary molecular and biomarker-based strategies have emerged in recent years to improve early detection of candidemia and invasive aspergillosis. These include nucleic-acid amplification platforms that bypass conventional DNA extraction, such as the T2Candida magnetic resonance assay, which has shown high clinical sensitivity in multicenter studies [12,13], and commercial real-time PCR assays targeting fungal ribosomal RNA gene regions, which have demonstrated good agreement with blood culture and galactomannan testing in recent clinical evaluations [14,15,16]. Despite these advances, non-culture diagnostics still require validation across diverse epidemiological settings, and data from Latin America remain particularly scarce [13].

This study aimed to evaluate the performance of the Immuno-Mycologics, Inc. (IMMY) CandID Multiplex PCR for the detection of *Candida* species in bloodstream infections, and the AspID Real-Time PCR for diagnosing invasive aspergillosis in patient populations at risk in Paraguay.

## 2. Materials and Methods

This study involved two patient cohorts: one for the evaluation of methods for the diagnosis of candidemia, and the second cohort for the diagnosis of invasive aspergillosis. The CandID assay targets the fungal ITS1/5.8S/ITS2 ribosomal RNA gene region—the standard universal fungal barcoding locus—enabling detection and species-level differentiation of the clinically relevant *Candida* species included in the panel. The AspID assay targets the 18S ribosomal RNA gene of *Aspergillus* spp. Both assays are commercial, manufacturer-validated kits, performed strictly according to the manufacturer’s package insert; primer, probe, and amplicon sequences are proprietary to the manufacturer and are not disclosed, consistent with reporting practice for other commercial molecular assays evaluated in clinical performance studies.

CandID Multiplex PCR Evaluation (Cohort A): From November 2023 to August 2024, we prospectively enrolled hospitalized patients with risk factors for *Candida* BSI from six medical institutions in Paraguay. Risk factors included leukemia, abdominal surgery, septic shock, diabetes, autoimmune diseases, kidney failure, dialysis, and broad-spectrum antibiotic use. Paired blood cultures (BC) and whole blood specimens for CandID Multiplex PCR testing were collected from each patient.

IMMY AspID Real-Time PCR (Cohort B): A second cohort of hospitalized patients at risk of IA was recruited from nine medical institutions in Paraguay between March 2024 and July 2024. Risk factors for IA included SOT, stem cell transplantation, leukemia, neuroblastoma, chronic liver disease, uncontrolled diabetes, myelodysplastic syndrome, and prolonged glucocorticoid therapy. Serum, plasma, and bronchoalveolar lavage (BAL) specimens were collected from patients with clinical suspicion of IA.

Specimens were tested for galactomannan antigen using the IMMY sōna *Aspergillus* GM lateral flow assay (GM-LFA) and for *Aspergillus* DNA using the IMMY AspID Real-Time PCR kit. 

DNA extraction: For both molecular assays, DNA extraction was carried out using the automated MagCore^®^ Genomic DNA extraction kits (RBC Bioscience, New Taipei City, Taiwan) preceded by a standardized specimen pretreatment step to enhance fungal cell disruption. For whole blood specimens used in the CandID assay, 200 µL of whole blood was mixed with 200 µL of lysis buffer, 20 µL of proteinase K, and zirconium beads in an Eppendorf tube. The mixture was vortexed, incubated at 56 °C for 10 min, and then vortexed again for an additional 10 min. After pretreatment, 400 µL of the lysate was transferred and supplemented with 10 µL of carrier solution before being loaded into the MagCore^®^ extraction cartridge. For the AspID assay, plasma specimens were processed using the same pretreatment procedure as whole blood. For bronchoalveolar lavage specimens, specimens were first centrifuged at 4000 rpm for 5 min, and 200 µL of the resulting sediment was then subjected to the same lysis, proteinase K digestion, bead-assisted disruption, and carrier-supplemented extraction procedure. Automated DNA extraction was performed with the elution volume set to 50 µL for all specimen types.

Real-time PCR amplification for both CandID and AspID assays was performed on the CFX96 Real-Time PCR Detection System (Bio-Rad, USA), according to the manufacturers’ instructions. The CandID Multiplex assay included two master mixes: one for the detection of *Candida albicans*, *C. glabrata* (*Nakaseomyces glabratus*), and *C. parapsilosis*, and another for *C. tropicalis*, *C. krusei* (*Pichia kudriavzevii*), and *C. dubliniensis*. Both reactions included an internal control to assess extraction/amplification performance and potential PCR inhibition. The CandID cycling protocol consisted of an initial denaturation at 95 °C for 2 min, followed by 40 cycles of 95 °C for 5 s and 60 °C for 20 s, with fluorescence acquisition during the 60 °C step. The AspID assay used a master mix for the detection of *Aspergillus* spp., also including an internal control. Its cycling protocol consisted of an initial denaturation at 95 °C for 3 min, followed by 40 cycles of 95 °C for 15 s and 63 °C for 60 s, with fluorescence acquisition during the 63 °C step. For both assays, each run included positive and negative controls, and results were interpreted only when the corresponding controls were valid. specimens with Ct values ≤38 were considered positive for the corresponding fungal target.

For patients who tested positive by the CandID Multiplex PCR or the AspID Real-Time PCR, a retrospective review of the electronic medical records was performed to confirm or discard a clinical diagnosis of invasive candidiasis or invasive aspergillosis, respectively. Diagnostic classification followed the revised EORTC/MSGERC consensus definitions of invasive fungal disease [17], complemented by current disease-specific clinical diagnostic guidance from the global ECMM/ISHAM/ASM candidiasis guideline and the ESCMID-ECMM-ERS guideline for the diagnosis and management of *Aspergillus* diseases [17,18,19,20].

Statistical analysis included descriptive metrics and agreement assessment using Cohen’s kappa. To evaluate the relationship between fungal burden markers, serum galactomannan optical density index (GM ODI) values were plotted against the corresponding *Aspergillus* PCR cycle threshold (Ct) values for each specimen. A simple linear regression model was fitted to characterize the association between the two variables, and the coefficient of determination (R^2^) and Pearson correlation coefficient (R) were calculated to quantify the strength and direction of the relationship. The 95% confidence bands around the regression line were generated to illustrate the precision of the model across the observed range of GM ODI values. Statistical significance was defined as *p* < 0.05. Analyses were performed using MedCalc. 

This manuscript was edited with the assistance of ChatGPT-5.5 and Claude Opus 4.

## 3. Results

Cohort A, CandID Multiplex PCR Performance: Thirty patients at risk of candidemia were included in the CandID evaluation. Forty-three percent were male; patient age ranged from 2 to 95 years (median: 62.5 years). Twelve patients (40%) had recent broad-spectrum antibiotic exposure. The CandID assay detected *Candida* DNA in seven out of 30 patients (23%), while blood cultures were positive in five out of 30 patients (17%), all patients with positive blood culture tested positive by the CandID PCR.

**Figure 1:**
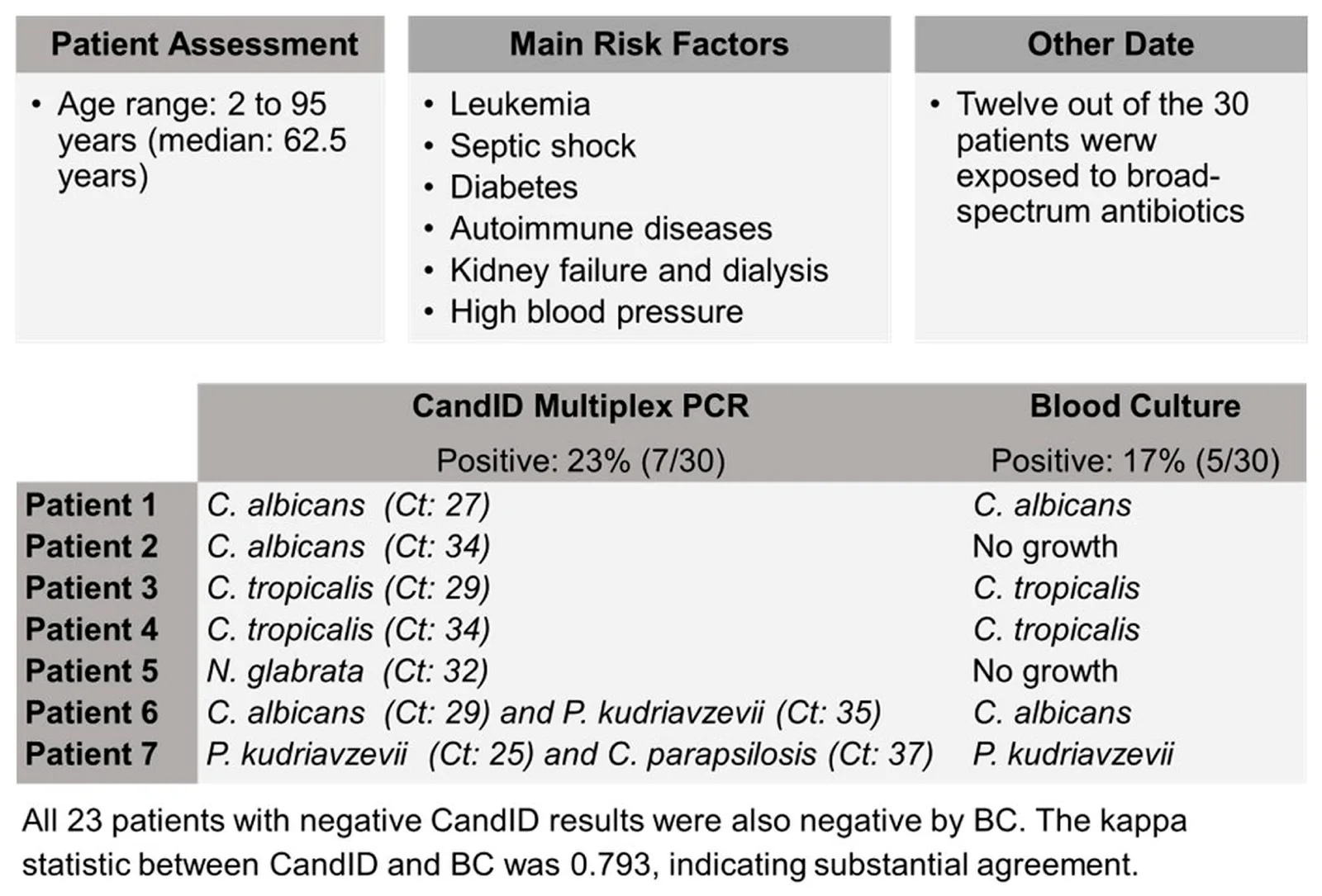
Results of the cohort A (n = 30), CandID multiplex PCR performance. All 23 patients who tested negative by CandID were also BC-negative. The kappa statistics measuring agreement between CandID and BC was 0.793, indicating substantial agreement.

In total, two patients had positive *C. albicans* PCR results (Ct values of 27 and 34), one of whom also had a positive BC confirming *C. albicans*. Two additional patients tested positive for *C. tropicalis* by PCR (Ct 29 and 34), both of whom also had positive BC for *C. tropicalis*. One patient demonstrated a positive PCR for *N. glabrata* (former *C. glabrata*) (Ct 32) with a negative BC. Two patients had two *Candida* species detected by PCR: one with *C. albicans* (Ct 29) and *P. kudriavzevii* (Former *C. krusei*) (Ct 35), with BC confirming *C. albicans*; and another with *P. kudriavzevii* (Ct 25) and *C. parapsilosis* (Ct 37), with BC positive for *P. kudriavzevii* (Figure 1).

Cohort B, AspID Real-Time PCR Performance: Twenty-nine patients were enrolled. Sixty-two percent were male; patient age ranged from 1 to 73 years (median of 33 years). Clinical presentations included prolonged fever, respiratory distress syndrome, and pulmonary infiltrates.

**Figure 2:**
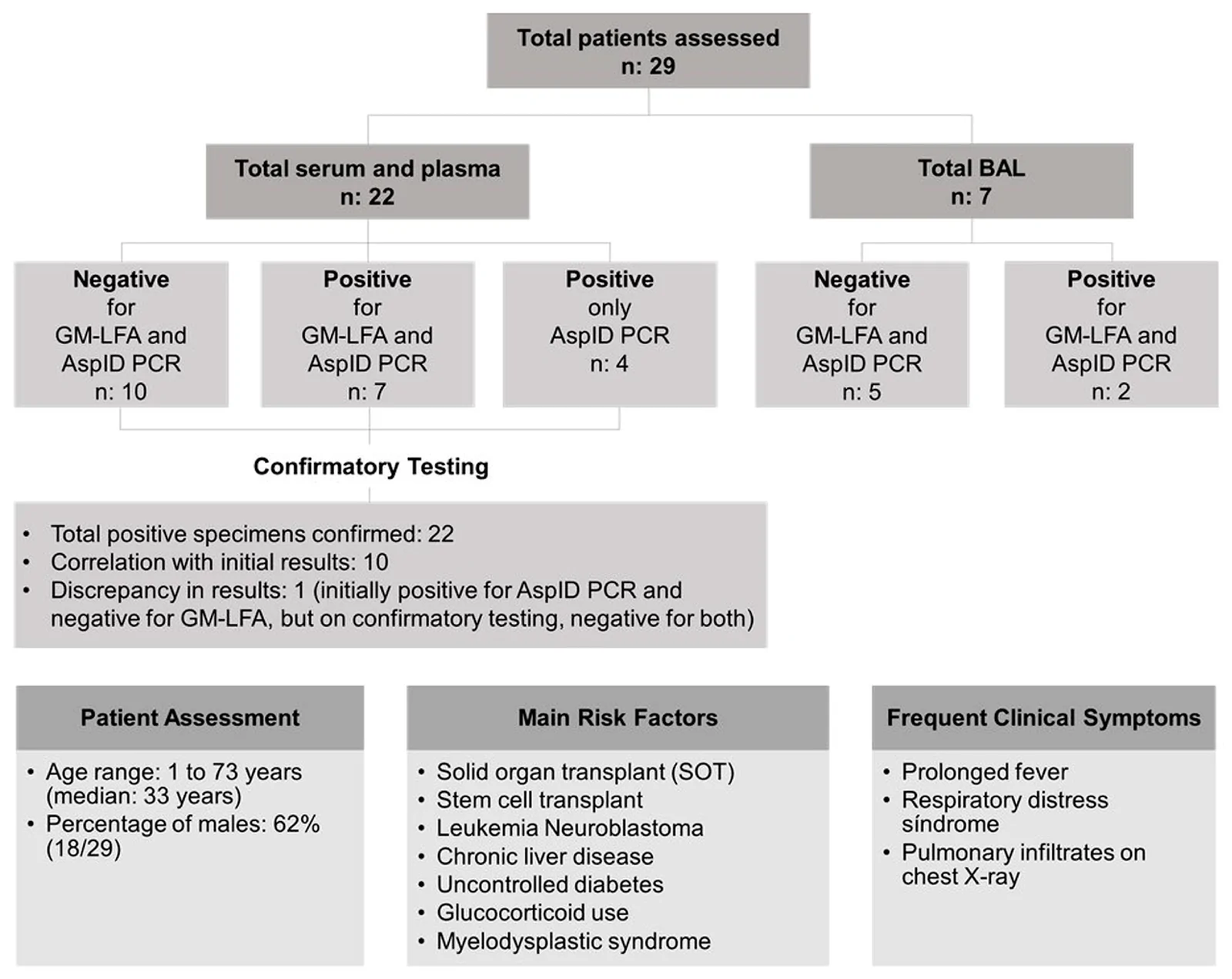
Results of the cohort B (n = 29), AspID real-time PCR performance.

A total of 22 serum/plasma specimens and 7 bronchoalveolar lavage (BAL) specimens were analyzed. Among the serum/plasma specimens, 10 were negative for both the GM-LFA and AspID PCR assays, while 7 tested positive by both methods. An additional four specimens were positive only by AspID PCR. Confirmatory testing yielded concordant results in all but one case, in which an initial AspID PCR-positive and GM-LFA-negative result was not confirmed upon repeat testing. Among the BAL specimens, 5 were negative for both assays, and 2 were positive for both GM-LFA and AspID PCR. All specimens that tested positive by PCR showed amplification for *Aspergillus* spp.; however, no specific amplification corresponding to *Aspergillus terreus* was observed (Figure 2).

**Figure 3:**
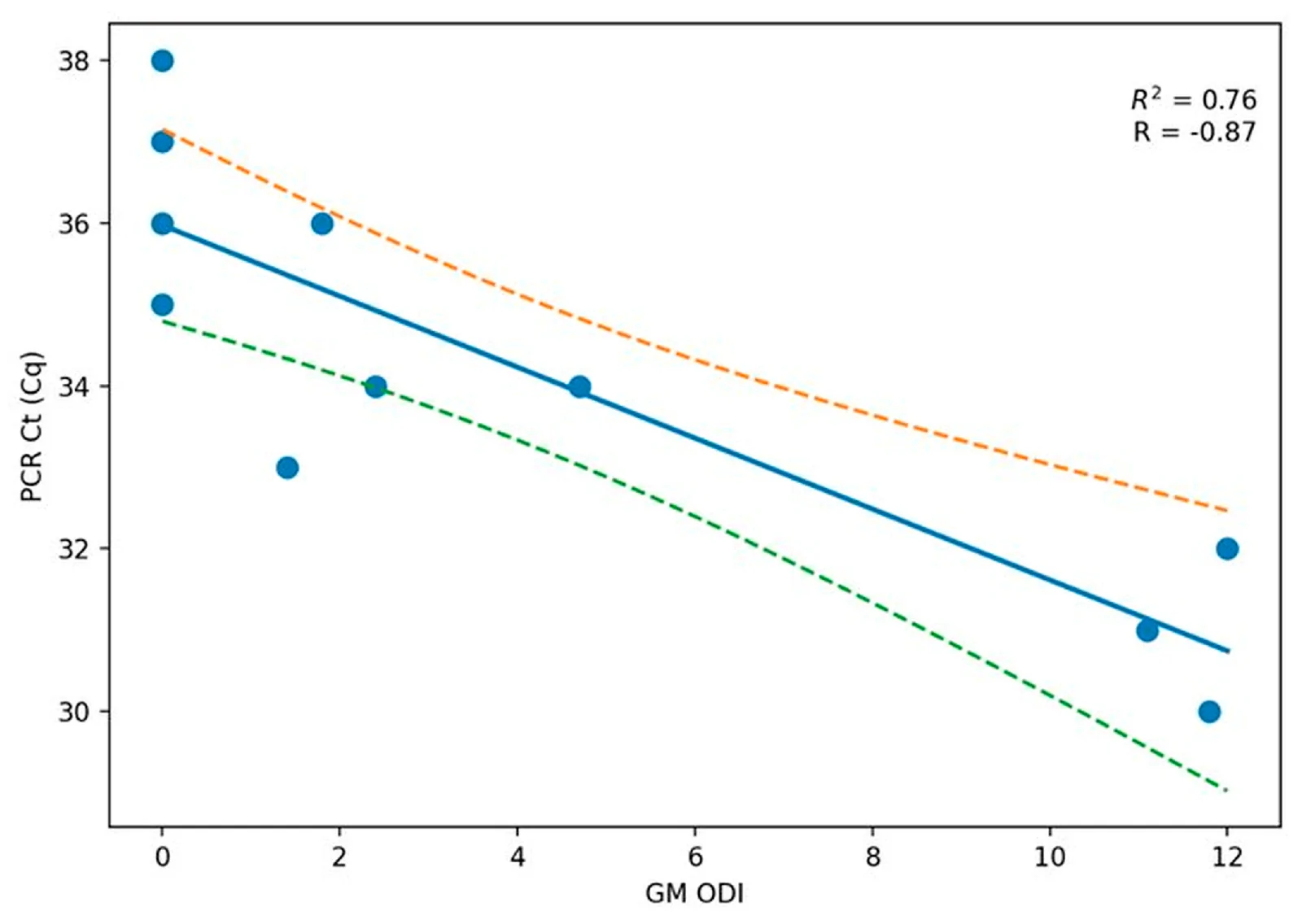
Inverse relationship between serum galactomannan (GM) levels and *Aspergillus* PCR cycle threshold (Ct). Scatter plot of GM ODI versus PCR Ct values, with fitted linear regression (solid line) and 95% confidence bands (dashed lines). A strong inverse correlation was observed (R^2^ = 0.76; R = −0.87), indicating that higher GM levels corresponded to lower Ct values, reflecting greater fungal burden.

To explore the relationship between fungal antigen burden and circulating fungal DNA in serum, we evaluated the association between galactomannan concentration (GM OD) and *Aspergillus* PCR Ct values. Across the eleven serum specimens with positive PCR, GM OD and PCR Ct demonstrated a strong inverse association (R = −0.87, R^2^ = 0.76), indicating that higher GM OD values were associated with lower Ct values. This finding is biologically consistent with the expectation that increasing circulating galactomannan levels reflect greater fungal burden, resulting in earlier PCR amplification. A linear regression model was fitted with Ct as the dependent variable and GM OD as the independent variable, and the fitted regression line with 95% confidence bands supported this relationship (Figure 3).

Because GM-negative specimens clustered at OD = 0.0 and could influence the correlation structure, a sensitivity analysis was performed including only GM-positive specimens (OD > 0.5; n = 7). This analysis confirmed a similar inverse association (R = −0.84, R^2^ = 0.70), supporting concordance between both biomarkers even when negative specimens were excluded.

Patient-level diagnoses, antifungal treatment, and 30-day outcomes: A retrospective review of the medical records of patients who tested positive by CandID and AspID confirmed that all had a clinical diagnosis of candidiasis or aspergillosis and received systemic antifungal therapy. Detailed information regarding antifungal treatment and 30-day patient outcomes is provided below and summarized in Table 1 and Table 2.

Among the seven patients diagnosed with candidemia by CandID Multiplex PCR, individual species identification, antifungal management, and outcomes were evaluated in detail. CandID detected two cases of *Candida albicans*, two cases of *Candida tropicalis*, one case of *N. glabrata*, and two mixed infections involving more than one *Candida* species. Blood cultures confirmed candidemia in five of these seven patients, while two patients were blood culture–negative but PCR-positive (Table 1).

Clinical management varied according to species detected and clinical evolution. The patient with *N. glabrata* detected only by PCR (Ct 32) and negative blood culture received fluconazole therapy but died on the seventh day of treatment. Two patients with *C. albicans* received fluconazole for 16 and 20 days, respectively, and both were discharged; one of these cases had blood culture confirmation. Two patients with *C. tropicalis* had concordant blood culture results; one was treated with fluconazole and died on day 14 of therapy, whereas the other received amphotericin B deoxycholate and died on day 6. One patient with simultaneous detection of *C. albicans* and *P. kudriavzevii* had blood culture confirmation of *C. albicans*, was treated with caspofungin, and survived to discharge. Another patient with *P. kudriavzevii* and *C. parapsilosis* detected by PCR had blood culture positive for *P. kudriavzevii*, received sequential fluconazole followed by caspofungin therapy, and was discharged. Overall, four of the seven patients survived to discharge, while three died during antifungal treatment (Table 1).

Thirteen patients were diagnosed with invasive aspergillosis by AspID PCR. All positive PCR specimens showed amplification for *Aspergillus* spp., without specific amplification for *Aspergillus terreus*. Antifungal regimens, duration of therapy, and 30-day outcomes were analyzed to contextualize the clinical relevance of molecular detection (Table 2).

Five patients initially received amphotericin B deoxycholate. Four of these patients died within the first 15 days of therapy. One patient was switched early to voriconazole but still died on day 15 of treatment. In contrast, one patient who received 15 days of amphotericin B followed by prolonged voriconazole therapy survived and was discharged. Three patients received liposomal amphotericin B as initial therapy; one of them died within 10 days, while the two patients who transitioned to voriconazole survived. Five patients received primary voriconazole-based therapy, and four of them survived to discharge after prolonged treatment courses ranging from two to three months. Only one patient treated with voriconazole died on day 10 of therapy. In total, seven of the thirteen patients survived to discharge, whereas four died early during antifungal treatment, predominantly among those who received amphotericin B deoxycholate as initial therapy (Table 2).

## 4. Discussion

As both CandID and AspID are already-validated commercial assays, this study was designed as a clinical performance evaluation in a real-world Paraguayan cohort rather than the analytical development of a new molecular test; the Discussion is accordingly focused on clinical concordance and patient outcomes rather than assay design. In this study, we evaluated the performance of two molecular assays in distinct patient cohorts, the CandID multiplex PCR for the detection of candidemia and the AspID real-time PCR for the diagnosis of invasive aspergillosis. 

The CandID assay demonstrated a higher detection rate (23%) compared to conventional blood cultures (17%) for candidemia, aligning with previous reports that highlight the limitations of culture-based methods due to their lower sensitivity and delayed turnaround times. Notably, all blood culture–positive cases were also detected by CandID PCR, reinforcing its potential role as a complementary diagnostic tool in clinical settings. These findings are consistent with the results reported by Trovato et al., who evaluated the use of the CandID Real-Time PCR in an ICU surveillance setting and confirmed its enhanced diagnostic yield over blood cultures, especially in patients with invasive candidiasis where early detection is critical [14]. Similarly, Price et al. demonstrated that CandID, when applied to serum specimens, improved diagnostic accuracy and showed promise as a non-culture-based assay for early detection [15]. Similar concordance between real-time PCR and blood culture, together with detection of dual candidemia missed by culture, has also been reported using an in-house qPCR assay in an ICU cohort [21], and comparably high species-level agreement (99%) between PCR performed directly on positive blood-culture bottles and conventional laboratory identification has been described with the CandID-PCR platform, supporting the added diagnostic value of rapid molecular speciation [22].

Interestingly, in two patients, *C. albicans* and *N. glabrata* DNA were detected by PCR despite negative blood cultures, suggesting possible early or low-level fungemia that may not have been captured by culture, or the presence of circulating fungal DNA from non-viable organisms. Additionally, in two other patients, PCR detected DNA from multiple *Candida* species. The ability of CandID to detect multiple *Candida* species within individual patients underscores its added value in providing species-level identification directly from clinical specimens—an advantage that may support timely and targeted antifungal therapy, especially in critically ill populations where delays in diagnosis are associated with poor outcomes. Notably, blood culture results correlated with PCR findings, and the species isolated by culture corresponded to the one with the lowest Ct value in PCR, indicating a higher fungal burden for that species. The strong agreement between CandID PCR and blood culture, reflected by a kappa value of 0.793, supports the assay’s reliability while also emphasizing its ability to identify additional cases that may be missed by culture alone.

In the evaluation of the AspID real-time PCR, our findings demonstrated a high level of concordance with the Galactomannan Lateral Flow Assay (GM-LFA), particularly in serum/plasma and BAL specimens. Among serum/plasma specimens, seven tested positive by both assays, while ten were negative by both, indicating good overall agreement. The detection of four additional PCR-positive cases with initially negative GM-LFA results may reflect the higher analytical sensitivity of molecular methods or the presence of early-stage infections with low circulating galactomannan levels. Only one discrepancy was observed upon confirmatory testing, supporting the high reproducibility of the AspID assay. In BAL specimens, agreement remained strong, with two positive and five negative results across both methods.

These findings are consistent with previous reports evaluating PCR assays and galactomannan detection tools. In a comparative study by Ghazanfari et al. [23], a strong correlation was observed between GM-LFA and GM-EIA in both serum and BAL samples from critically ill patients with suspected invasive pulmonary aspergillosis. In that study, GM-LFA in serum demonstrated a sensitivity of 56% and specificity of 94%, while in BAL, sensitivity and specificity were 60.6% and 88.9%, respectively.

Similarly, Chowdhury et al. evaluated both commercial and in-house *Aspergillus* PCR assays in BAL specimens from patients with chronic pulmonary aspergillosis (CPA). Although PCR showed moderate sensitivity (~52%), its performance varied depending on clinical context and specimen type [24]. The authors emphasized the value of using PCR in combination with galactomannan assays to enhance diagnostic accuracy, particularly when interpreted alongside clinical and radiological findings.

The observed strong inverse association between serum galactomannan concentration and *Aspergillus* PCR Ct values provides evidence of concordance between circulating antigen levels and fungal DNA signal in this cohort. This relationship is biologically plausible, as increasing galactomannan concentrations are expected to reflect higher fungal burden, which in turn facilitates earlier PCR amplification and lower Ct values. The consistency of this association, demonstrated both in the full dataset and in the sensitivity analysis restricted to GM-positive specimens, suggests that the correlation is not driven solely by the clustering of GM-negative specimens at OD = 0.0. The linear regression model and accompanying confidence bands further support the stability of this trend across the observed range of antigen concentrations. Together, these findings indicate that serum GM intensity and PCR Ct values may provide complementary and biologically aligned information regarding fungal burden, reinforcing the potential value of integrating both biomarkers in the interpretation of invasive aspergillosis diagnostics.

Patient-level diagnoses, antifungal treatment, and 30-day outcomes provided additional clinical context to the diagnostic performance of both assays. A retrospective review of the medical records of patients who tested positive by CandID and AspID confirmed that all had a clinical diagnosis of candidiasis or aspergillosis and received systemic antifungal therapy. Among the seven patients diagnosed with candidemia by CandID PCR, species identification directly influenced antifungal selection and was associated with heterogeneous clinical outcomes. Four patients survived to discharge, while three died during antifungal therapy. Importantly, two of the fatal cases occurred in patients with BC-confirmed *C. tropicalis* infection treated with fluconazole or amphotericin B deoxycholate, whereas patients with *C. albicans* and mixed infections who received caspofungin or prolonged fluconazole therapy survived. In the aspergillosis cohort, nine of thirteen patients survived to discharge. Mortality was predominantly observed among patients who received amphotericin B deoxycholate as initial therapy, whereas most patients managed with primary or early voriconazole-based regimens survived after prolonged treatment courses. This pattern mirrors the landmark randomized trial establishing voriconazole’s survival advantage over conventional amphotericin B deoxycholate as primary therapy for invasive aspergillosis [25].

These findings highlight the complementary role of molecular assays in the diagnosis of invasive fungal infections, particularly in settings where rapid and accurate detection is essential for timely initiation of appropriate antifungal therapy. Both the CandID and AspID PCR assays demonstrated high diagnostic concordance with established methods, while offering potential advantages in sensitivity and species-level identification, which is critical for guiding appropriate antifungal therapy given the variable susceptibility profiles among different *Candida* and *Aspergillus* species.

Importantly, both assays also proved feasible and clinically relevant in a resource-limited setting, such as Paraguay, where conventional culture methods are often limited by delayed turnaround times and restricted access to advanced diagnostic platforms. This is consistent with regional surveys documenting persistent gaps in mycology diagnostic capacity across Latin America and the Caribbean [26], and with the recent WHO landscape analysis identifying molecular diagnostics as one of the most limited resources for invasive fungal disease in low- and middle-income countries [27]. Further studies involving larger cohorts and comprehensive clinical correlation are warranted to validate these preliminary observations and to better define the role of these molecular tools in routine clinical practice.

This study has important limitations. The most relevant limitation is the small sample size in both cohorts, which restricts the statistical power of the analysis and limits the generalizability of the findings. As both CandID and AspID are commercial, already-validated diagnostic kits, this study was designed as a direct clinical application/evaluation of these assays rather than the development or in-house analytical validation of a new molecular test; generating a calibration curve, determining analytical sensitivity (limit of detection), or performing dedicated cross-reactivity panels (e.g., against *A. terreus*, *A. flavus*, *A. niger*) therefore fell outside the scope of this evaluation and was not performed by our group. Notably, all AspID-positive clinical specimens in our cohort showed amplification consistent with *Aspergillus* spp., with no amplification pattern specific to *A. terreus* observed. Within each cohort, the molecular assay and its comparator were performed on paired specimens from the same source (blood culture and CandID PCR on the same blood draw in Cohort A; GM-LFA and AspID PCR on the same serum/plasma or BAL specimen in Cohort B), so sample-source discordance does not explain the mismatched results observed within pairs. Patients who tested negative by both the molecular assay and its comparator were not classified as having candidiasis or aspergillosis, and detailed retrospective clinical review (diagnosis, antifungal treatment, and 30-day outcomes) was restricted to test-positive patients; double-negative patients were not systematically re-evaluated for a possible missed diagnosis at a site not represented by the sampled specimen (e.g., deep-seated or extravascular disease). This represents a form of verification bias inherent to designs where detailed clinical confirmation is limited to test-positive cases, and it should be considered when interpreting the reported diagnostic performance. The patient-level outcome analysis should therefore be interpreted with caution, as the number of events is insufficient to establish causal relationships between diagnostic methods, antifungal choice, and clinical outcome. In addition, the retrospective nature of the clinical data review may have introduced information bias, and the absence of standardized treatment protocols across patients may have influenced outcomes independently of diagnostic timing. Further studies involving larger patient cohorts, prospective designs, and standardized clinical management are warranted to validate these preliminary observations and to better define the role of these molecular tools in routine clinical practice.

## 5. Conclusions

This study represents the first clinical evaluation of the CandID and AspID molecular assays in Latin America. Both assays provided rapid, sensitive, and clinically relevant information for the diagnosis of invasive candidiasis and aspergillosis, respectively. Integration of molecular assays with conventional diagnostics enhances early detection, allows for prompt targeted therapy, and may ultimately improve outcomes in patients with invasive fungal infections.

## Figures and Tables

**Table 1:** Patient-level diagnoses, antifungal treatment regimens, and 30-day outcomes in patients with candidemia and who tested positive by CandID.

	CandID Multiplex PCR Result	Blood Culture Result	Candidemia Diagnosis (Yes/No)	Candidemia Treatment Antifungal/Doses/Duration	30 Days Outcome
**Patient 1**	*N. glabrata* (Ct: 32)	No growth	Yes	Fluconazole 800 mg on day 1, then 400 mg/day for 6 days	Died on day 7 of treatment
**Patient 2**	*C. albicans* (Ct: 34)	No growth	Yes	Fluconazole 800 mg on day 1, then 400 mg/day for 16 days	Discharged
**Patient 3**	*C. albicans* (Ct: 27)	*C. albicans*	Yes	Fluconazole 800 mg on day 1, then 400 mg/day for 20 days	Discharged
**Patient 4**	*C. tropicalis* (Ct: 29)	*C. tropicalis*	Yes	Fluconazole 400 mg/day for 14 days	Died on day 14 of treatment
**Patient 5**	*C. tropicalis* (Ct: 34)	*C. tropicalis*	Yes	Amphotericin B deoxycholate, total dose 400 mg	Died on day 6 of treatment
**Patient 6**	*C. albicans* (Ct: 29) and *P. kudriavzevii* (Ct: 35)	*C. albicans*	Yes	Caspofungin 70 mg on day 1, then 50 mg/day for 15 days	Discharged
**Patient 7**	*P. kudriavzevii* (Ct: 25) and *C. parapsilosis* (Ct: 37)	*P. kudriavzevii*	Yes	Fluconazole 400 mg/day for the first 3 days, then caspofungin 70 mg on day 1, followed by 50 mg/day for 16 days	Discharged

**Table 2:** Patient-level diagnoses of invasive aspergillosis (IA), antifungal treatment regimens, and 30-day outcomes in patients with invasive aspergillosis who tested positive by AspID.

	AspID PCR Result	*Aspergillus* GM LFA	IA Diagnosis (Yes/No)	IA Treatment Antifungal/Doses/Duration)	30 Days Outcome
**Patient 1**	Positive sera (Ct: 34)	Positive sera (index: 4.7)	Yes	Amphotericin B deoxycholate 0.7 mg/kg/day for 14 days	Died on day 14 of treatment
**Patient 2**	Positive sera (Ct: 32)	Positive sera (index: 12.0)	Yes	Amphotericin B deoxycholate 0.7 mg/kg/day for 12 days	Died on day 12 of treatment
**Patient 3**	Positive sera (Ct: 30)	Positive sera (index: 11.8)	Yes	Amphotericin B deoxycholate 0.7 mg/kg/day for 10 days	Died on day 10 of treatment
**Patient 4**	Positive sera (Ct: 33)	Positive sera (index: 1.4)	Yes	Amphotericin B deoxycholate 0.7 mg/kg/day for 4 days, then voriconazole 4 mg/kg every 12 h	Died on day 15 of treatment
**Patient 5**	Positive sera (Ct: 31)	Positive sera (index: 11.1)	Yes	Voriconazole 6 mg/kg every 12 h on day 1, then 4 mg/kg every 12 h for 3 months	Discharged
**Patient 6**	Positive sera (Ct: 34)	Positive sera (index: 2.4)	Yes	Liposomal amphotericin B 3 mg/kg/day for 10 days	Died on day 10 of treatment
**Patient 7**	Positive sera (Ct: 36)	Positive sera (index: 1.8)	Yes	Voriconazole 6 mg/kg every 12 h on day 1, then 4 mg/kg every 12 h for 2.5 months	Discharged
**Patient 8**	Positive sera (Ct: 38)	Negative sera	Yes	Voriconazole 6 mg/kg every 12 h on day 1, then 4 mg/kg every 12 h for 2 months	Discharged
**Patient 9**	Positive sera (Ct: 35)	Negative sera	Yes	Amphotericin B deoxycholate 0.7 mg/kg/day for 15 days, then voriconazole 4 mg/kg every 12 h for 3 months	Discharged
**Patient 10**	Positive sera (Ct: 36)	Negative sera	Yes	Liposomal amphotericin B 3 mg/kg/day for 15 days, then voriconazole 4 mg/kg every 12 h for 2 months	Discharged
**Patient 11**	Positive sera (Ct: 37)	Negative sera	Yes	Voriconazole 6 mg/kg every 12 h on day 1, then 4 mg/kg every 12 h	Died on day 10 of treatment
**Patient 12**	Positive BAL (Ct: 35)	Positive BAL (index: 1.8)	Yes	Liposomal amphotericin B 3 mg/kg/day for 5 days, then voriconazole 4 mg/kg every 12 h for 2.5 months	Discharged
**Patient 13**	Positive BAL (Ct: 32)	Positive BAL (index: 0.5)	Yes	Voriconazole 6 mg/kg every 12 h on day 1, then 4 mg/kg every 12 h for 2 months	Discharged

## Data Availability

The data presented in this study are available on request from the corresponding author or first authors.
